# Spontaneous omental bleeding: a case report and literature review

**DOI:** 10.1186/s12893-018-0364-9

**Published:** 2018-05-30

**Authors:** Yun-Xiao Lyu, Yun-Xiao Cheng, Ting Li

**Affiliations:** 1 Department of Hepatobiliary Surgery, Dongyang People’s Hospital, No. 60, West Wuning Road, Dongyang, Jinhua, Zhejiang China; 2 Dongyang People’s Hospital, No. 60, West Wuning Road, Dongyang, Jinhua, Zhejiang China; 3 Department of General Surgery, Dongyang People’s Hospital, No. 60, West Wuning Road, Dongyang, 322100 Zhejiang Province China

**Keywords:** Omental bleeding, Diagnosis, Computerd tomography, Surgery, Transcatheter arterial embolization

## Abstract

**Background:**

Spontaneous rupture of omental vessels is an infrequent medical condition possibly causing severe intra-abdominal hemorrhage. Omental bleeding results from trauma associated injury and irritation, neoplasia, arterial aneurysm rupture, and anticoagulant treatment. Idiopathic omental bleeding rarely causes acute abdominal bleeding which has been reported to occur in previous studies. Here we reported a case with idiopathic omental hemorrhage due to vascular malformation. A systematic review of literature is provided.

**Case presentation:**

A 58-year-old Han Chinese man arrived at the emergency department with left upper quadrant abdominal pain for 1 day. He had no significant previous medical history. There was no history of fever, vomiting, nausea, or anorexia. He was a non-smoker and did not consume alcohol. On physical examination, blood pressure was 118/72 mmHg, for a temperature of 37.7 °C; heart and respiratory rates of 130 per/min and 20 per/min were obtained, respectively. Abdomen assessment showed only mild tenderness in the left upper quadrant. Complete blood count (CBC) showed white cell and platelet counts of 16.69 × 10^3^/L and 196 × 10^3^/L, respectively. The haemoglobin value was 13.5 g/L at admission. Abdominal Computer Tomography (CT) was performed that showed peritoneal fluid appeared around the liver. Fresh blood was confirmed in the abdominocentesis. A hemoperitoneum was confirmed by abdominal enhanced CT, which presented a structural disorder in the left upper abdomen. The subject immediately underwent exploratory laparotomy. A massive hemoperitoneum originating from omental vessels was observed. The omental were partially removed. There was no evidence of malignancy or aneurysm upon palpation. Pathological assessment of the extracted tissue pointed to vascular malformation. The patient subsequently had an uneventful recovery; hospital discharge occurred at 7 days post-operation.

Previous reports assessing idiopathic omental bleeding were systematically reviewed, summarizing published cases. A total of 12 hits were found in PubMed for idiopathic omental bleeding.

**Conclusion:**

Idiopathic omental bleeding is a rare condition that requires emergency treatment. Treatment strategies include surgical intervention and transcatheter arterial embolization (TAE). The surgical option is suitable in subjects with persistent hypotension and those with unconfirmed diagnosis.

## Background

Spontaneous rupture of omental vessels is an infrequent medical condition which causes serious intra-abdominal bleeding. Omental bleeding can result from trauma associated injury and irritation, neoplasia [1], arterial aneurysm rupture [2], and treatment with anticoagulants [3]. Idiopathic omental bleeding rarely causes acute abdominal bleeding which has been reported to occur in previous studies. Here, we reported a case with idiopathic omental hemorrhage due to vascular malformation. In addition, previous reports were systematically reviewed.

## Case presentation

**Fig. 1:**
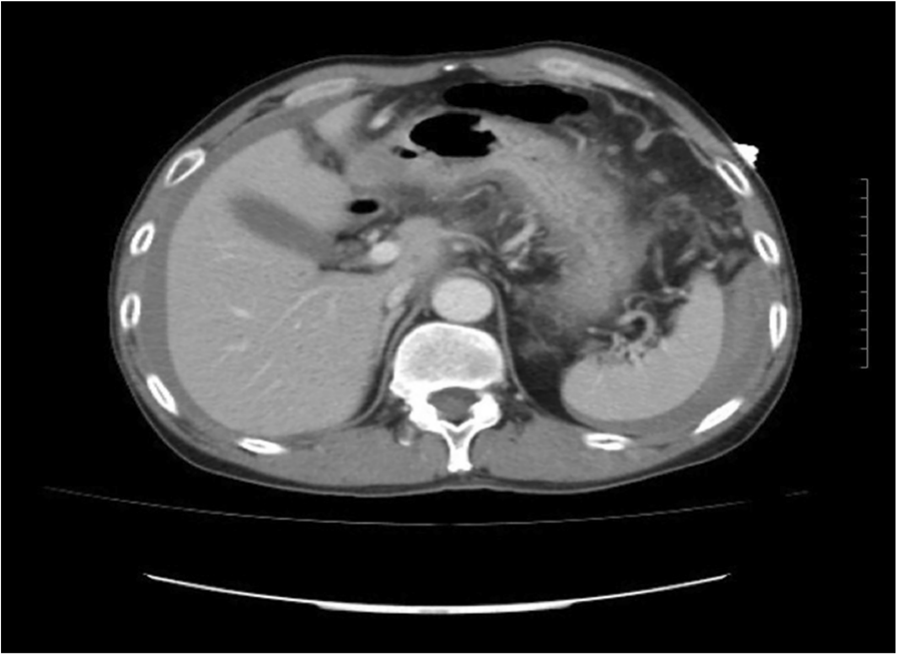
Abdominal CT scan. The CT-scan receals structural disorder of the left upper abdominal and hemoperitoneum

**Fig. 2:**
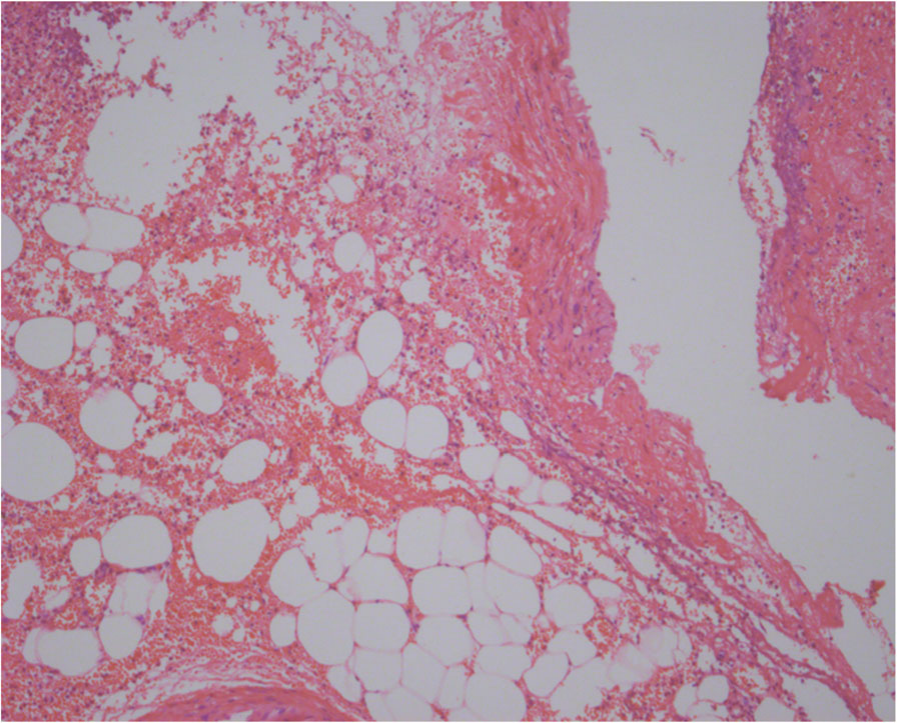
Pathology examination. The pathology reveals the the vascular malformations of omental

A 58-year-old Han Chinese man arrived at the emergency department with left upper quadrant abdominal pain for 1 day. He had no significant previous medical history. There was no history of fever, vomiting, nausea, or anorexia. He was a non-smoker and did not consume alcohol. On physical examination, blood pressure was 118/72 mmHg, for a temperature of 37.7 °C; heart and respiratory rates of 130 per/min and 20 per/min were obtained, respectively. Abdomen assessment showed only mild tenderness in the left upper quadrant. Complete blood count (CBC) showed white cell and platelet counts of 16.69 × 10^3^/L and 196 × 10^3^/L, respectively. The haemoglobin value was 13.5 g/L at admission. Abdominal Computer Tomography (CT) was performed that showed peritoneal fluid. In order to clarify the nature of peritoneal effusion,abdominocentesis was performed. Fresh blood was confirmed in the abdominocentesis. We could not identify the source of bleeding through abdominal CT. A hemoperitoneum was confirmed by abdominal CT with contrast enhancement, which presented a structural disorder in the left upper abdomen (Fig. 1). The subject immediately underwent exploratory laparotomy. A massive hemoperitoneum originating from omental vessels was observed. The omental were partially removed. There was no evidence of malignancy or aneurysm upon palpation. Pathological assessment of the extracted tissue pointed to vascular malformation(Fig. 2). Our pathologist found that it is venous malformation with the damage of the venous wall continuity accompanied with the overflow of large number of red blood cells. The patient subsequently had an uneventful recovery; hospital discharge occurred at 7 days post-operation.

Previous reports assessing idiopathic omental bleeding were systematically reviewed, summarizing published cases. A total of 12 hits were found in PubMed for idiopathic omental bleeding.

### Review of the literature

The PubMed (2000–2017) database was queried for case reports of idiopathic omental bleeding. The abstracts of all articles published in the English language were screened. Patient data, including age, diagnostic and treatment procedures, were extracted.

A total of 12 articles were found in PubMed for idiopathic omental bleeding, including ours [4–15]. Relevant findings are summarized in Table 1. The patients included 11 males and 1 female, aged between 20 and 70 years. The diagnostic procedures included CT and laparotomy. The patients underwent emergency surgery (*n* = 8) or transcatheter arterial embolization(TAE) (*n* = 4).

**Table 1:** Reports of idiopathic omental hemorrhag

First of author	Year	Coutry	F/M	Age	Chief complaint	Post medical history	Diagnostic procedure	Treatment
Kroot EJ [7]	2003	Netherlands	M	70y	Abdominal pain	NA	CT	Surgery
Finely DS [5]	2005	USA	M	41y	Abdominal pain Difficulty seeing	Alcoholic cirrhosis (took a unkonwn dose of sidenafi)	Hemoglobin drop	Ligate the omental varix
Ohno T [10]	2005	Japan	M	27y	Intermittent abdominal pain	Surgery for cryptorchidism	CT	Partial omentalectomy
Jadav M [6]	2004	USA	M	60y	Acute abdominal pain Nausea,voimiting and diarrha	Hypertension	laparotomy	Surgery
Nagaba Y [9]	2005	Japan	M	64y	Acute abdominal pain hemorrhagic shock	Autosomal-dominant polycystic kidney disease	CT	TAE
Tsuchiya R [12]	2009	Japan	M	58y	abdominal pain	NA	CT	TAE
Matsumoto T [8]	2010	Japan	M	25y	Abdominal pain	NA	CT	TAE
Henry D [13]	2012	USA	F	24y	malaise, myalgias, and fatigue	NA	laparotomy	Surgery
Takahashi M [11]	2012	Japan	M	27y	abdominal pain. temporary loss of consciousness	NA	CT	TAE
Cheng VE [4]	2014	Australia	M	68y	acutely hypotensive with severe left sided abdominal pain	Inferior STEMI ticagrelor and aspirin	CT	Partial omentectomy
Aumann V [14]	2016	Germany	M	20	NA	Hemophilia A	NA	Surgery
Kimura J [15]	2016	Japan	M	29	Abdominal pain	NA	CT	Partial omentectomy

*NA* not avaliable, *CT* computer tomography, *TAE* transcatheter arterial embolization

## Discussion and conclusions

Idiopathic omental bleeding, although sparse in this part of the world, is considered one of the causes of spontaneous hemoperitoneum. Spontaneous omental bleeding is a serious condition, with a mortality rate exceeding 30% [16]. Several causes of spontaneous omental bleeding have been reported, including neoplasia, arterial aneurysm, vasculitis, and anticoagulant therapy. A patient administered sildenafil citrate succumbed to the rupture of an omental varix [5]. However, there are few reports of idiopathic omental bleeding. The ages of patients with idiopathic omental bleeding range between the 20s and the 80s; it has a male predominance. Acute intraabdominal hemorrhage, abdominal pain and distension, tachycardia, and hypotension, constitute typical signs of idiopathic omental bleeding; severe cases present with abdominal compartment syndrome [17]. Some cases series assessing omental bleeding suspected appendicitis or peritonitis preoperatively [10, 18]. The diagnostic assessment of idiopathic omental bleeding is essentially based on imaging procedures, especially ultrasonography (US) and CT. US facilitated hemoperitoneum detection in the current hemodynamically unstable subject. US is considered as an effective method. However, in our hospital, US needs to be done by a professional ultrasound-doctor. However, CT (especially enhanced CT) is the most effective imaging tool since signals corresponding to hemoperitoneum, active arterial extravasation, and mesenteric fluid might help radiologists determine the origin of hemorrhage and guide treatment [16]. Abdominocentesis can be a useful diagnostic tool in distinguishing the characteristic of peritoneal fluid. However, abdominocentesis is an invasive procedure which can be lead to intestinal perforation and abdominal wall abscess. When the patient’s condition is unstable, it may be appropriate to have a laparotomy or a laparoscopy.

Regardless of the underlying etiology of idiopathic omental bleeding, aggressive treatment is preferable. Idiopathic omental is routinely treated by surgical procedures, including ligation or omentectomy. In most cases reported, however, an emergency surgery was performed. The surgical option is suitable in subjects with persistent hypotension and those with unconfirmed diagnosis. Surgery is often carried out because few cases are correctly diagnosed pre-treatment. However, TAE for idiopathic omental bleeding has been reported previously [8, 9, 11, 12]. TAE is a safe and minimally invasive procedure, with the advantages of simultaneous diagnosis and treatment. Therefore, TAE might represent the best therapeutic option for idiopathic omental bleeding. It should be carried out with caution in patients with proximal embolization due to risk of rebleeding via the collateral circulation. A therapeutic scheme is proposed for the treatment of idiopathic omental bleeding in this study.

In summary, idiopathic omental bleeding is an infrequent condition requiring emergency treatment; typical manifestations include acute intraperitoneal hemorrhage. US and CT scan are useful for its diagnosis. Treatment strategies include surgical intervention and TAE. In subjects with persistent hypotension or cases with unconfirmed diagnosis, surgery might be suitable.

## Abbreviations

CT: Computed Tomography
TAE: Transcatheter arterial embolization
US: Ultrasound
